# The Hospital World

**Published:** 1910-09-10

**Authors:** 


### The Hospital World.

### Elections and Appointments.

Miss Nicholson has been appointed lady superintendent at the Manchester Children's Hospital. She has been on the nursing staff of St. Bartholomew's.

Sir Hugh Blackett, Lord Joicey, the Right Hon. Thomas Burt, Mr. W. D. Cruddas, Councillor W. J. Sanderson, Dr. Hume, and Mr. Thomas Buckan have been appointed vice-presidents of the Newcastle Royal Infirmary; and Sir George Hare Philipson, Mr. J. T. Patton, Mr. C. Francis Lloyd, and Mr. James B. Clark have been appointed hon. life governors.

Mr. Alderman J. Collinson has been re-elected President of the Halifax Eye, Ear, and Throat · Hospital. The following gentlemen were also re-elected: Hon. Treasurer, Mr. A. R. Pollit; Hon. Auditor, Mr. W. H. Mitchell; Hon. Secretary, Mr. F. Mitchell; the Committee, Canon Ivens, Mr. W. Rothwell (Stainland), Mr. W. S. Milligan, Mr. Edwin Turner, Mr. George Thwaite, Mr. James Butler, Mr. John Marsh, Mr. McCormack, Mr. L. Marshall, Mr. R. P. Hattersley, and Mr. Hugh Campbell.

### Personalia.

By the death of Mr. George Watson Smyth, C.B., the Royal Waterloo Hospital for Children and Women has sustained a great loss. For the past six years he has been actively associated with this institution as a member of the board of governors and as hon. secretary. "It is seldom," remarks a friend, in the obituary notice in the *Times*, "that an institution has the privilege of being served so loyally by a vigorous man in the full maturity of his wisdom and judgment." The numerous letters of sympathy which have been received testify to the esteem in which he was held by all who had intercourse with him in the affairs of the hospital.

The death is announced from Walton Lodge, Chesterfield, of Captain William Wynne Jeudwine, who had been agent for the Dukes of Devonshire's Chesterfield, Staveley, Hardwick, Dore, and Lincoln estates for forty years. He was born in 1842, and was the son of the Rev. William Jeudwine, vicar of Chicheley, Bucks. Captain Jeudwine was known in the district as one of the men who gave much personal service to the cause of the sick, and who took a great interest in charitable institutions. He was for some years Chairman of the Chesterfield and North Derbyshire Hospital, and worked assiduously to promote the welfare of the institution.

The death was announced last week of Miss Catherine Drew, who for over twenty years had been engaged in journalistic work in London, and who, during her lifetime, was a staunch friend of charitable work. She was a sister of the late Sir Thomas Drew, President of the Royal Hibernian Academy, was of Irish birth, and at the time of her death was a vice-president of the Institute of Journalists. For many years she devoted her energies to the work of strengthening the finances and enlarging the scope of the Orphan Fund of the Institute. Her pen was always at the service of charity, and many an institution owes a debt of gratitude to her for her support and assistance, always cheerfully and readily given.

By the death of Alderman John Pretyman Slingsby Roberts, the Brighton hospitals lose a staunch friend. It is now over thirty years since Mr. Roberts left London, where he practised as a solicitor, and went to Brighton. There he interested himself quickly in institutional work, and became Vice-President of the Sussex County Hospital. His interest in the hospitals of his town was of the most practical kind, and his knowledge of what institutional work implies was proved fully by his election to the post of Honorary Secretary to the Throat and Ear Hospital. A man of many activities, his political energies and the more ceremonial duties of his high office among the Freemasons of Sussex, of whom he was Provincial Grand Secretary, never detracted from his labours for the hospitals. Indeed, the many sides of life with which he was acquainted—for instance, as solicitor, Freemason, and Mayor—gave to his work for hospitals a peculiar value. To that work he brought a knowledge of men and affairs which a less active life would have made impossible. To the Brighton hospitals his loss will be a very real one.

Among the many distinguished institutional workers in London who were held in peculiar respect, the late Mr. H. A. Harben, once chairman, and till his death treasurer and trustee, of St. Mary's Hospital, held a high place. The record of his work for St. Mary's is inspiring in more ways than one. There were few institutional posts connected with his hospital that at one time or another he had not held, and few capacities as a benefactor towards it which he did not fill. His energies and his banking account were alike ready to be drawn upon in time of need. In 1892 he became a subscriber, and future annual reports will have an air of omission about them without his well-known name that for eighteen years had figured there so regularly. In 1897 he entered into the full tide of hospital work by his election to the board of management, and so well was his work appreciated that his election as a vice-president four years later was the stepping-stone to his appointment as chairman in 1903. As vice-president he gave £500 and obtained £1,650 for the Clarence Wing Fund towards securing the Zunz Bequest. In 1902 he gave £3,300 to free the medical school from debt. These two munificent gifts were only the first of a list of benefactions that extended till his death in August. Thus the second year of his chairmanship—1904—was marked by a further present of £2,000 from himself, and of £5,000 from his sister, Mrs. Wharrie, towards the medical school. Next year the confidence and regard he inspired in his colleagues led to his appointment as treasurer and trustee, and in 1906 he saved the medical school again by his generosity. A terrazzo pavement for the ground floor of the old hospital was badly wanted, and so was the repainting of its walls, when Mr. Harben stepped forward and provided the means for obtaining them. In the midst of his generous schemes of such wide scope as those we have mentioned, Mr. Harben did not forget details, and the piano in the students' common room is a present from him. His goodwill towards the school he had done so much for already never lessened, and in 1907 a new system of drainage was installed at his expense. The sisters, too, have to thank him for his care for their wants in fitting up a dining-room for them. This was one of his last acts before resigning the chairmanship, which he did in 1909, and the six years during which he was in office will not easily be forgotten. Through good report and evil report, and St. Mary's, like other great institutions, has had anxious as well as prosperous times, Mr. Harben was a constant and powerful friend. For eighteen years he gave of his best, his energy, his brains, and his money, and his record of work in the short outline here given is one of which all institutional workers, outside as well as inside his hospital, must be proud.

